# Copy Number Variation Screen Identifies a Rare *De Novo* Deletion at Chromosome 15q13.1-13.3 in a Child with Language Impairment

**DOI:** 10.1371/journal.pone.0134997

**Published:** 2015-08-11

**Authors:** Kerry A. Pettigrew, Emily Reeves, Ruth Leavett, Marianna E. Hayiou-Thomas, Anahita Sharma, Nuala H. Simpson, Angela Martinelli, Paul Thompson, Charles Hulme, Margaret J. Snowling, Dianne F. Newbury, Silvia Paracchini

**Affiliations:** 1 School of Medicine, University of St Andrews, St Andrews, United Kingdom; 2 Department of Psychology, University of York, York, United Kingdom; 3 Wellcome Trust Centre for Human Genetics, University of Oxford, Oxford, United Kingdom; 4 Department of Experimental Psychology, University of Oxford, Oxford, United Kingdom; 5 Division of Psychology and Language Sciences, University College London, London, United Kingdom; 6 St. Johns College, University of Oxford, Oxford, United Kingdom; University of Leuven, BELGIUM

## Abstract

A significant proportion of children (up to 7% in the UK) present with pronounced language difficulties that cannot be explained by obvious causes like other neurological and medical conditions. A substantial genetic component is predicted to underlie such language problems. Copy number variants (CNVs) have been implicated in neurodevelopmental and psychiatric conditions, such as autism and schizophrenia, but it is not fully established to what extent they might contribute to language disorders. We conducted a CNV screen in a longitudinal cohort of young children with language-related difficulties (n = 85), focusing on single events at candidate loci. We detected a *de novo* deletion on chromosome 15q13.1–13.3. The adjacent 15q11-13.1 locus is disrupted in Prader-Willi and Angelman syndromes, while disruptions across the breakpoints (BP1-BP6) have previously been implicated in different neurodevelopmental phenotypes including autism, intellectual disability (ID), seizures and developmental delay (DD). This is the first report of a deletion at BP3-BP5 being linked to a deficit confined to language impairment, in the absence of ID, expanding the range of phenotypes that implicate the chromosome 15q13 locus.

## Introduction

Approximately 3–7% of pre-school English-speaking children have pronounced language impairment (LI) [1], which cannot be accounted for by intellectual, sensory or physical impairment, or by poor educational opportunities [2,3]. LI is often the manifestation of other profound deficits or conditions. Furthermore, it often co-occurs with learning difficulties, such as reading problems (or dyslexia). [4,5], attention deficit/hyperactivity disorder (ADHD) [6,7] and autism spectrum disorder (ASD)[8,9]. These observations exemplify the challenges of defining clear and distinct diagnostic criteria for disorders that could in fact be different manifestations along a spectrum of a core deficit and might share some common genetic determinants [3].

Family and twin studies indicate a substantial genetic influence in LI [10], estimating heritability to be around 70%, with a complex multifactorial mode of inheritance. Genetic associations reported so far explain only a small fraction of the estimated heritability [11]. Common variants have been identified mainly through association studies at specific loci, including the *CNTNAP2*, *CMIP* and *ATP2C2* genes[11]. A few genome-wide association studies (GWAS) for language abilities have been conducted in epidemiological samples [11]. The most significant finding has been the association between *ROBO2* and expressive vocabulary in young children[12] The only locus reaching genome-wide significance in clinical samples collected for LI was reported at the *NOP9* locus, when modelling for parent-of-origin effects [13]. A few chromosomal rearrangements have also been identified. *SEMA6D*, a gene located on chromosome 15q21, has been implicated in language difficulties by breakpoint mapping of a balanced duplication [14].An emerging role for copy number variants (CNV) in disease aetiology has been reported for several neurodevelopmental and psychiatric disorders [15]. Rare deletions and duplications have been implicated in intellectual disability (ID), developmental delay (DD) and autism spectrum disorder (ASD) [16–18], all of which can manifest language problems. Several studies have shown a greater CNV burden of large events in ID, DD and ASD cases compared with unaffected individuals [18,19].

In addition to overall CNV burden across the genome as a whole, recurrent rearrangements at specific loci are often associated with syndromes characterised by speech and language delay in conjunction with other specific features. Examples include the 22q13 deletion syndrome [20], Smith-Magenis syndrome caused by deletions at 17p11 [21] and Potocki-Lupski syndrome (PTLS), caused by a duplication at chromosome 17p11.2 [22], presenting with language delay and speech difficulties.

Disruptions at 15q11-q13.3 have been implicated in different neurodevelopmental phenotypes depending on the locations of breakpoints. Recurrent chromosomal rearrangements at this locus occur as a result of non-allelic homologous recombination between six breakpoints (BP1-BP6) (S1 Fig), corresponding to regions containing multiple copies of the human chromosome 15 low-copy repeat (LCR15) duplicon [23]. While deletions at BP2-BP3 occur in Prader-Willi (PWS) [24] and Angelman syndromes (AS) [25], BP4-BP5 deletions and duplications are associated with a range of neuropsychiatric outcomes such as ID/DD, epilepsy, speech problems, ASD, schizophrenia, mood disorder and ADHD[26].

To date, very few studies have assessed the contribution of CNVs to LI. One CNV screening, in a cohort of children with LI, identified a microdeletion in the *ZNF277* gene in an individual with severe receptive and expressive language impairment. This microdeletion was inherited from both parents, who were heterozygous and had a history of language difficulties [27]. Further analysis of this locus in an additional 321 families selected for LI identified another 5 carriers of the microdeletions, showing an increased frequency (1.1%) compared to control cohorts (0.4%). Genome-wide burden analysis in 152 families selected for LI implicated inherited common CNVs, not as single events, but in combination with other genetic risk factors [28]. However, *de novo* CNV appeared to be relevant in simplex families.

Here, we report a screen for CNV events in 85 children selected for having language difficulties and/or a family risk of reading problems from a longitudinal study of language and literacy development. We report a *de novo* BP3-BP5 deletion at chromosome 15q13.1–13.3 in a child with early-diagnosed language impairment. This is the first report of a 15q13 deletion at the BP3-BP5 breakpoints to be implicated in LI with no other apparent medical conditions or generalised developmental problems.

## Materials and Methods

### Samples

Eighty-six individuals (n = 64 probands, n = 22 siblings) were selected from a sample of children participating in a longitudinal study of reading and language development. The 64 probands were recruited because either showed preschool language impairment and/or had a family risk of dyslexia (FRD) [29]. As expected, many children with preschool language impairment resolved their spoken language difficulties by age 5. At the time of entering the genetic screening, when children were 8–9 years old, 44 children were classified as having language impairment and/or dyslexia and the remaining 42 children were classified as typically developing.

The cohort has been described previously [29,30] and further details are provided in S1 File. Exclusionary criteria included chronic illness, deafness, English as a second language or known neurological disorder (e.g. epilepsy, cerebral palsy, ASD).

Saliva was collected from probands, siblings and parents using the Oragene Collection kit and DNA extracted according to the manufacturer’s instructions (DNA Genotek Inc, Ottawa, Canada). DNA from parents and unscreened siblings was available for validation experiments to determine whether CNVs were *de novo* or inherited. Classification of probands’ status according to language, reading and mathematical abilities was determined using assessments undertaken during the course of the study. Confirmatory factor analysis was performed using the Mplus software [31] in order to condense these individual assessments to seven constructs at pre-school and school age (S1 Table). Additional details were available, including age of first words, age of first walking, birth weight and medical conditions. The study was approved by NHS Research Ethics (Yorkshire and the Humber—Humber Bridge) and the University of York, Department of Psychology Ethics Committee. Written consent was obtained from all study participants or from the parents or guardians on behalf of the children enrolled in this study.

### Copy Number Variation screening

All 86 children were genotyped using the Illumina Human OmniExpress-24 platform which includes ~700,000 SNPs. Analysis was conducted with Illumina GenomeStudio 2011 (Genotyping module v1.9.4, Genome Viewer v1.9.0). Any SNP with a GenTrain (internal quality) score of < 0.5 or genotyping success rate of < 95% was excluded from further analyses. One individual was excluded because genotype data indicated a possible contamination. The chromosome plots of all individuals were examined manually to detect any large copy number events. CNVs were predicted from SNP data within PennCNV (2011 Jun16 version) [32] and QuantiSNP v2.2 [33] packages. All individuals had a standard deviation (SD) for the LogR ratio < 0.35, a B-allele frequency drift < 0.002 and waviness factor within ± 0.04 in PennCNV analyses, and an average LogR ratio SD < 0.3 and B-allele frequency SD < 0.15 in QuantiSNP. Consistent high confidence CNVs were selected, based on the following criteria: spanning at least three consecutive genetic variants, confidence score > 10, and predicted by both algorithms. The consistent high confidence CNV calls were then compared with those reported in control individuals (Database of Genomic Variants (DGV), version GRCh37/hg19, release date 16/10/2014) and were allocated to the following categories: (i) common (overlapping ≥ 50% with ≥ 5 events in the control database), (ii) rare (overlapping ≥ 50% with < 5 events), (iii) rare with low overlap (overlapping with known events but < 50%) and (iv) novel (not overlapping with any known event).

### CNV validation

We selected CNVs for validation that were either rare or novel (according to the (ii), (iii) and (iv) categories described above) and disrupted loci previously implicated in neurodevelopmental conditions or disorders presenting symptoms of language difficulties. We prioritised rare or novel events recurring in our cohort at higher frequency than expected and events that spanned all or part of at least one exon. Selected CNVs were validated by quantitative PCR (qPCR) in a 10μl reaction volume containing genomic DNA, 1X SsoFast EvaGreen supermix (Bio-Rad Laboratories Ltd, Hertfordshire, UK) and an assay-dependent amount of each primer. Primer sequences are detailed in S2 Table. Reactions were cycled using a Viia7 instrument (Life Technologies, Paisley, UK), for 40 cycles with an annealing temperature of 64°C. Melt curve analysis was performed, showing a single clear peak for each assay. Relative quantification of the amplicons was calculated based on comparison with a known two-copy control fragment, using the 2-ΔΔCt method [34]. All assays were conducted in at least three replicates. Analysis of confirmed CNVs was extended to all available family members both by qPCR and genome-wide genotyping using the Illumina Human OmniExpress platform. The latter data were used to test for relatedness across family members and checking for Medelian errors, and to infer the parental chromosome on which the deletion originated.

## Results and Discussion

### CNV screening

Of the 86 individuals genotyped with the Omni-Express Illumina platform, 85 passed standard quality control criteria. QuantiSNP predicted individual CNVs of size ranging from 306 bp to 828 kb, while PennCNV predicted a size range of 306 bp to 1.86 Mb (S3 and S4 Tables). In order to ensure detection of all possible events, low detection thresholds were applied (confidence levels > 10 and spanning ≥ 3 SNPs). In total, 3169 consistent high-confidence CNVs were identified, of which 89.1% were duplications (S5 Table). A full list of all consistent high-confidence CNVs identified in probands and siblings has been submitted to the DGV archive (http://www.ebi.ac.uk/dgva; accession number estd220).

On average, 37.28 CNVs were detected per individual. Depending on the predictive algorithm used, 26–41% of CNVs were larger than 100 kb in size, and 4–12% larger than 200 kb (S4 Table). Individual CNV sizes mentioned in this study are consensus predictions of both algorithms. The majority of detected CNVs (88%, n = 2795) were located within at least one known gene, and 36.1% of those (n = 1010) were rare or novel CNVs.

Because of the relatively small size of our cohort we focused our analysis on individual large events rather than comprehensive CNV assessment, such as burden analysis. Three CNVs were selected for follow-up and validation by qPCR because of their occurrence at loci previously reported to be associated with neurodevelopmental phenotypes in the literature. These included a deletion at chromosome 15q13.1–13.3, which spanned 3.08 Mb, consisting of two adjacent deletions of 1.86 Mb and 868.7 kb separated by a repetitive region. Other events were selected for investigation but did not validate. These included a duplication (789.2 kb) at chromosome 16q13.11, and two overlapping duplications spanning exon 1 of the Semaphorin 3A (*SEMA3A)* gene predicted in two unrelated probands (64.6 kb and 45.7 kb).

The chromosome 15q deletion was the largest event to be identified in this cohort. The next largest event (> 1.5 Mb; S4 Table) was a duplication at chromosome 14, a common CNV, previously reported in DGV.

### Chromosome 15q13.1–13.3 deletion

**Fig 1:**
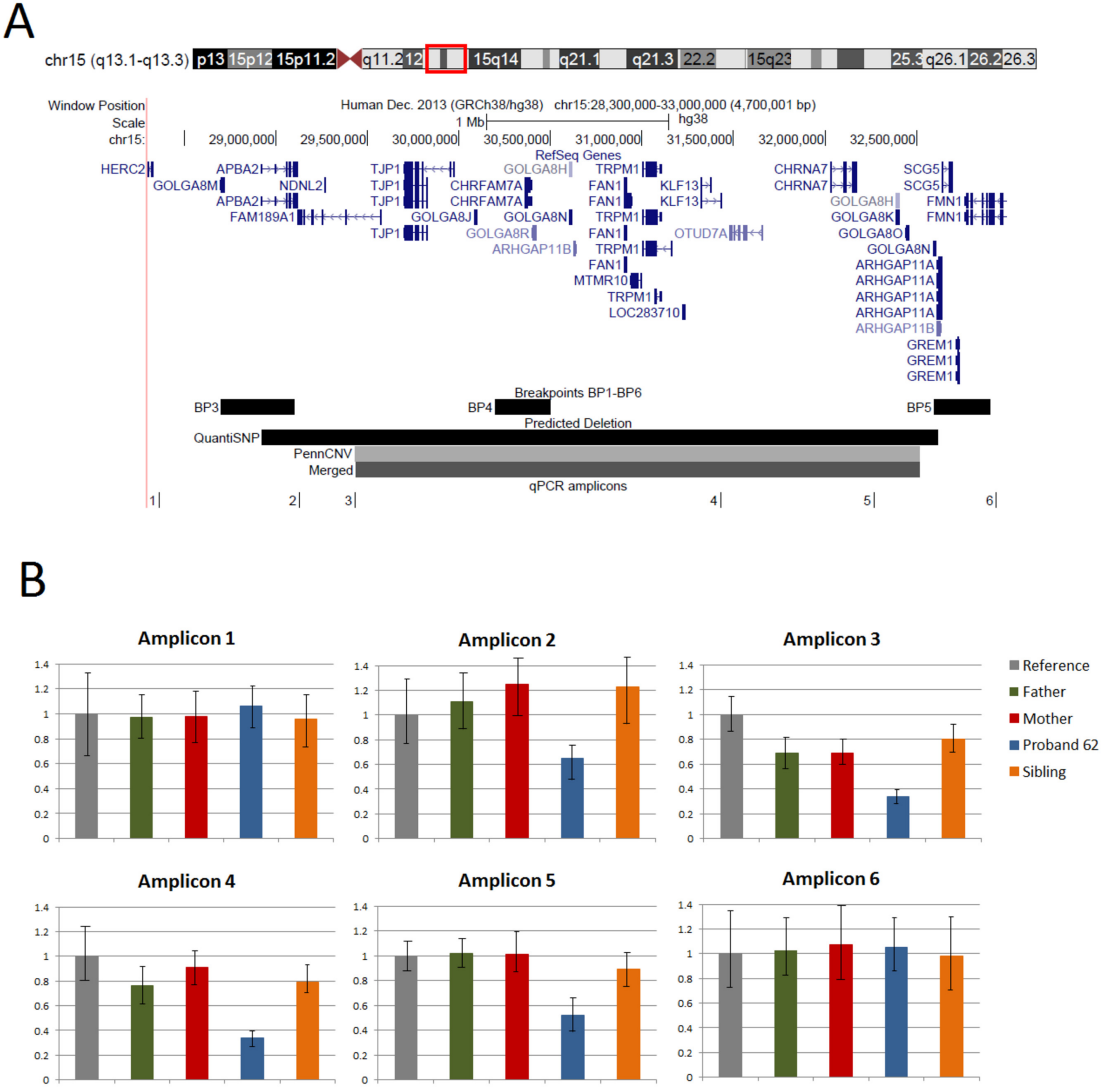
Deletion at the chr15q13.1–13.3 locus in a language impaired proband. (A) A snapshot from the UCSC Genome Browser (http://genome.ucsc.edu/; hg19) showing the genomic region encompassed by the chr15q13.1–13.3 deletion identified in a child with language impairment. The tracks at the bottom indicate the breakpoints previously mapped at this region (BP3; BP4 and BP5); the deletion predictions by QuantiSNP, PennCNV and by merging the two algorithms; and the location of the six amplicons used for validation. (B) qPCR results for the six amplicons in the proband’s family (colour coded bars indicate: father in green; mother in red; proband in blue and sibling in orange; the reference in grey). Approximately 50% reduction in copy number at amplicons 2–5 in the proband relative to reference and family members indicates a *de novo* deletion occurring at BP3-BP5.

We detected a BP3-BP5 deletion at chromosome 15q13.1–13.3 predicted to be 3.08 Mb in size (Fig 1A), in a child with language impairment (proband 62). The deletion was validated by qPCR (Fig 1B). Analysis in the unaffected family members (mother, father and sibling) by both qPCR and genome-wide genotyping clearly indicated that this event has a *de novo* origin. Genome-wide genotyped data confirmed the relatedness of the family members and showed that the deletion originated on the paternal chromosome. There were 94 mendelian errors out of 860 SNP analysed at the chromosome 15 locus all indicating lack of paternal alleles. In contrast, only 81 other errors were found across the rest of the genome and IBD (identical by descendent) sharing between parents and both siblings was 0.5 as expected.

The 15q11-13 locus is a highly unstable genomic region, which originates CNVs across six specific breakpoints (BP1-BP6) (S1 Fig). Deletions at the 15q11-13 locus have been implicated in several neurodevelopmental conditions. Prader-Willi (PWS) and Angelman (AS) syndromes most commonly occur as the result of deletions between BP2-BP3, although a more severe phenotype is observed for larger events across BP1-BP3 [24,25]. CNVs restricted to the BP1-BP2 region have been associated with reading and mathematical difficulties and general cognitive functioning [35]. Disruptions between BP4 and BP5 (15q13.2–13.3) have been reported in individuals with seizures and ID [36,37]. Larger BP3-BP5 deletions (15q13.1–13.3), similar to the CNV reported here, have only been observed in isolated cases presenting a variable phenotype (Table 1).

**Table 1:** Clinical characteristics of previously reported cases carrying a BP3-BP5 deletion.

Case	Clinical characteristics	Reference
1	Epilepsy, mild learning difficulties, lax thumb joint, squint, developmental delay	[36]
2	Severe learning difficulties, autism, moderate language delay	[38]
3	Epilepsy	[39]
4	Hypotonia, dyspraxia, autism	[40]
5	Epilepsy, learning difficulties, microcephaly, growth <3rd centile, reduced hemisphere volume, psychomotor retardation, optic nerve atrophy?	[41]
6	Low birth weight, natal teeth, heart murmur, microcephaly, delayed speech and walking, poor attention	[42]
7	Epilepsy, Mild learning difficulties	[43]
8	Moderate intellectual disability, obesity, ADHD, hypotonia, delayed speech, strabismus	[26]

Proband 62 was recruited to the study because of clinical concerns regarding speech and language development. Both the parents and sibling score above average on cognitive tests, and there is no family history of dyslexia or language impairment. The proband spoke the first words at 21 months (> 1 SD of the cohort mean age at first word) and presented at 3 years and 7 months with difficulty producing speech-sounds coupled with problems of expressive and receptive language development. The first steps of proband 62 occurred at 16 months (1 SD above the cohort mean age of first walking), and performance was poor on the Movement-ABC test for fine motor skills. A family history of motor problems was reported. Proband 62 was born prematurely after an induced birth, weighing just below 2.5 kg, but otherwise has no record of other medical problems that could explain these language difficulties.

Proband 62 was assessed five times at approximately annual intervals from age 3 years and 7 months to 7 years and 8 months (see S6 Table for scores on measures of non-verbal ability, language and literacy measures). There was some variation in levels of performance over time, as might be expected given that language and cognitive skills are less stable across this period of development than later in childhood. At the first time point (T1), the proband fulfilled criteria for language impairment and speech sound disorder. Generally the pattern was persistent and at 6 years some immature speech processes (e.g. fronting) were still present. Non-verbal IQ was above 70 throughout, indicating no evidence of intellectual disability but was notably lower at age 7.8 years than earlier in development. While the poorer performance may be due to poor attention, such a decline is often observed in children with LI [44]. Proband 62 made a slow start in learning to read, and phonological awareness remains an area of weakness, but reading and spelling skills are within the normal range. In summary, in early primary school, language impairment was present in the absence of literacy difficulties.

## Discussion

We conducted a CNV screen in 85 children recruited in a longitudinal cohort designed to study the development of children with impaired language and/or a family history of dyslexia [29,30]. Because our small sample is not adequate to conduct a comprehensive and generalised assessment of the role of CNVs in reading and language abilities at the burden level, we focused our analysis on single large events affecting loci previously implicated in neurodevelopmental phenotypes.

We identified a large *de novo* deletion at chromosome 15q13.1–13.3 in a child who at 3 ½ years presented with language impairment and comorbid speech-sound disorder. Symptoms of language impairment persisted through to the age of 7 years when speech difficulties were mostly resolved, but scores on non-verbal tasks had weakened. Literacy development was normal in all respects at the latest assessment. The deletion was confirmed to be between BP3 and BP5, spanning more than 3 Mb and at least 15 genes (Fig 1A and 1B).

To date, only a limited number of cases carrying a BP3-BP5 deletion have been reported, presenting a variety of symptoms including seizures and general neurodevelopmental impairment (Table 1). In addition to language problems, only weak executive motor skills were observed in the child described in the current study. The observation of a family history of motor control problems suggest this trait might not be the direct consequence of the deletion which has a *de novo* origin. Seizures and overt physical disability were exclusionary criteria for this study, and no medical conditions were reported since recruitment. To the best of our knowledge, this is the first report of a BP3-BP5 deletion in an individual with language impairment, normal reading skills and no evidence of sensory and neurological problems. Language difficulties are often considered as secondary effects of a broader condition, but our results suggest that haploinsufficiency of genes in the BP3-BP5 region can influence language ability directly, reinforcing the view that recurrent CNVs relevant for neurodevelopmental phenotypes tend to be incompletely penetrant and associated with a variable phenotype [45]. The BP3-BP5 deletion has not yet been reported in healthy controls, despite screenings in large cohorts. For example, Lowther et al. reported that none of the 23,838 adult controls screened for 15q13.3 deletion before 2014 presented a BP3-BP5 deletion[26]. The rarity of this event is further confirmed by the detection of only four carriers of BP3-BP5 deletions across 34, 046 individuals referred for clinical testing because of symptoms of mental retardation, developmental delay, or multiple congenital anomalies [42] (Table 1). Our results, however, can also be interpreted in the context of very limited CNV screenings in cohorts selected specifically for language impairments. Individuals with seizures or ID are more systematically assessed for CNV and, consequently, rare deletions such as at BP3-BP5 have a higher probability of being detected in these groups. A recent CNV screening in children with LI did not identify any events comparably large at the chromosome 15 locus, but did show a slightly higher frequency of rearrangement at this region compared to controls (15.7% *vs*. 13.8%); however, this difference was entirely driven by duplications (7.1% *vs*. 5.2%) [28]. By screening larger groups with LI it will be possible to assess the relevance of deletions at this locus, as well as other large chromosomal rearrangement, in contributing to language difficulties. This is consistent with our previous report showing a relatively higher frequency of sex chromosome aneuploidies in children with language impairment [46].

It is clear that many genetic factors still need to be identified to understand the genetic architecture underlying the aetiology of language difficulties, but we expect to see the same scenario that is emerging for most common and complex disorders, demonstrating the contributions of both rare and common variants [11]. In particular, resequencing technology is likely to identify many rare variants private to very few individuals that might explain language difficulties under a monogenic model.

The absence of the deletion in controls suggests that this event is sufficient to lead to a range of neurodevelopmental phenotypes, the severity of which might depend on additional factors. The rarity of the BP3-BP5 deletion, the broad spectrum of its clinical manifestations, and the large number of genes involved make it extremely difficult to hypothesise what molecular mechanisms might lead to language problems specifically. Genetic background and environmental factors are likely to account for much of the phenotypic variation observed between individuals carrying the same deletion. Parental origin could be another factor contributing to heterogeneity. Our analysis indicates that the deletion originated on the paternal chromosome. It would be interesting to systematically assess parental effects of previously reported BP3-BP5 deletions, but that is prevented by lack of data. It has been suggested that deletion of the *CHRNA7* gene alone, which was predicted to be deleted in the proband described in this study, might mediate most of the phenotypes associated to the BP4-BP5 deletions [47–49].

The medical history of proband 62 did not suggest any causal risk factors for language problems, except perhaps induced pre-term birth with low birth weight. It has been observed that near-term children, born at 33–37 weeks, have poorer educational outcomes and a higher incidence of neurological, behavioural and developmental disturbances than full-term children [50,51]. However, it is difficult to establish a causal link and it remains possible that an underlying genetic condition may increase the risk of developing obstetric complications and requiring induced birth.

From a technical point of view, we have shown that two of the three CNVs predicted by two algorithms were not validated by qPCR. Both CNVs were predicted to be duplications, so it is possible that complex chromosomal rearrangements have affected either the original prediction or the replication experiments. We accept that our results can still be considered inconclusive but, by reporting them, we highlight the necessity of validating predicted CNVs with alternative methods.

To summarise, we have identified a *de novo* deletion at chromosome 15q13.1–13.3 in a child with language impairment. This is the first report showing that a deletion between BP3-BP5 breakpoints affects language abilities directly rather than leading to a more general and profound phenotype. While there is some evidence that non-verbal abilities in proband 62 had fallen to just below the average range at 7 years, such a decline is not uncommon among children with SLI. In addition to extend the characterisation of the phenotypic manifestations of the chromosome 15q BP3-BP5 deletion, our results implicate for the first time the chromosome 15q13.1–13.3 locus in the aetiology of language difficulties, further expanding our understanding of the genetic risk factors underlying this condition.

## Supporting Information

S1 Fig UCSC Genome Browser snapshot (http://genome.ucsc.edu/; hg19) showing the chromosome 15q11-13 CNV region defined by breakpoints (BP) 1–6. (TIF)

S1 File Cohort Description and Assessment. (DOCX)

S1 Table Confirmatory factor analysis. (DOCX)

S2 Table Quantitative PCR primers. (DOCX)

S3 Table Predicted CNV sizes. (DOCX)

S4 Table Size distribution of predicted CNVs. (DOCX)

S5 Table CNVs frequency. (DOCX)

S6 Table Raw scores for performance of Proband 62. (DOCX)
