# Supplementary figures and images for: Copy Number Variation Screen Identifies a Rare De Novo Deletion at Chromosome 15q13.1-13.3 in a Child with Language Impairment
Source: PLoS One. 2015 Aug 11;10(8):e0134997. doi: 10.1371/journal.pone.0134997 (PMC4532445; doi:10.1371/journal.pone.0134997)

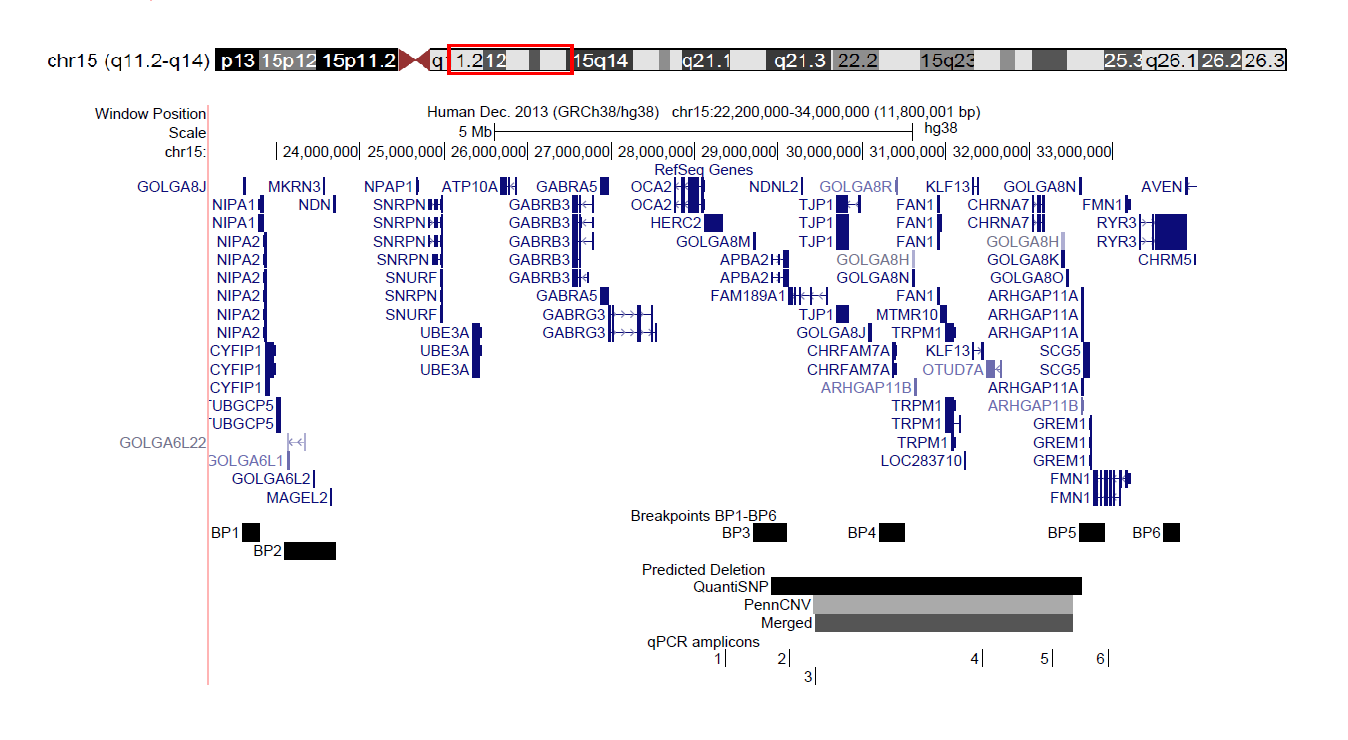

Supplement: S1 Fig — (TIF) [file pone.0134997.s001.tif]
