# Supplementary material for: Copy Number Variation Screen Identifies a Rare De Novo Deletion at Chromosome 15q13.1-13.3 in a Child with Language Impairment
Source: PLoS One. 2015 Aug 11;10(8):e0134997. doi: 10.1371/journal.pone.0134997 (PMC4532445; doi:10.1371/journal.pone.0134997)
Supplement: S1 File — (DOCX) [file pone.0134997.s002.docx]

**S1 File. Cohort Description and Assessment**

This cohort was recruited as part of a study of reading and language development [1,2].

Families with 3-year-old children were recruited through advertisement in local newspapers, nurseries and webpages aimed at individuals with dyslexia/language difficulties, and via Yorkshire services provided for speech and language therapy. Exclusion criteria included: monozygotic twins, chronic illness, deafness, a non-English first language, care provision by local authority and known neurological disorder such as cerebral palsy, epilepsy and autism spectrum disorders. The children were then classified into groups to determine whether or not they were at family risk for dyslexia (FRD) on the basis of parental questionnaire and assessment and whether they had current language impairment (LI). Of the 86 participants included in the present study, 46 probands and 22 siblings were at family risk for dyslexia (FRD), defined as having (i) a parent self-reporting reading difficulties, (ii) a parent scoring < 90 on a literacy composite of non-word reading and spelling, (iii) a parent with a ≥ 1.5 SD discrepancy between non-verbal ability and literacy composite, and with a literacy composite standard score ≤ 96 or (iv) a sibling diagnosed with dyslexia by an educational psychologist or specialist teacher [2]. The remaining participants without a FRD had a classification of language impairment at recruitment (mean age 3.8 years).

A child was classified as language impaired if scored ≤ 7 on two out of the four following tests: (i) Clinical Evaluation of Language Fundamentals (CELF) preschool [3] basic concepts, (ii) CELF expressive vocabulary, (iii) CELF sentence structure, and/or on one test and failed the Test of Early Grammatical Impairment (TEGI) [4] screener. Language ability was reassessed (mean age 8.1 years) as part of a larger assessment battery designed to identify dyslexia. Many children resolve their preschool LI. A reading and spelling composite score was calculated from the average of Single Word Reading (SWR) [5] and Wechsler Individual Achievement Test (WIAT) spelling scores [6]. A child was classified as having ‘dyslexia’ if this standardised score was less than 88.

The older siblings of children participating in the longitudinal study were also recruited. More details for this cohort have been reported previously [1]. Parents also underwent testing for reading, language and communication skills.

The cohort was assessed with a comprehensive range of tests assessing language and reading skills, as well as other cognitive traits relating to neurodevelopment. For the current study, 7 factors were used to assess language and reading abilities and were derived from a larger number of individual measures, both at pre-school and school age: (i) non-verbal IQ, (ii) speech, (iii) language grammar, (iv) language vocabulary, (v) literacy, (vi) phonology and (vii) rapid automatised naming (RAN). The contribution of individual tests to these factors is detailed in Supplementary Table 1. In addition, individual measures were used, such as “age at first word”, birth weight and reported medical conditions.

**References**

1. Moll K, Loff A, Snowling MJ. Cognitive Endophenotypes of Dyslexia. Sci Stud Read. 2013;17: 385-397.

2. Nash HM, Hulme C, Gooch D, Snowling MJ. Preschool language profiles of children at family risk of dyslexia: continuities with specific language impairment. J Child Psychol Psychiatry. 2013;54: 958-968.

3. Semel E, Wiig EH, Secord W. Clinical evaluation of language fundamentals - fourth edition UK (CELF-4UK). London: Harcourt Assessment; 2006.

4. Rice ML, Wexler K. Rice/Wexler test of early grammatical impairment. New York: Psychological Corporation; 2001.

5. Foster H. Single word reading test 6-16. London: GL Assessment Limited; 2007.

6. Wechsler D. Wechsler Individual Achievement Test–Second UK Edition (WIAT-II UK): London, UK: Psychological Corporation; 2005.
