# Supplementary material for: Copy Number Variation Screen Identifies a Rare De Novo Deletion at Chromosome 15q13.1-13.3 in a Child with Language Impairment
Source: PLoS One. 2015 Aug 11;10(8):e0134997. doi: 10.1371/journal.pone.0134997 (PMC4532445; doi:10.1371/journal.pone.0134997)
Supplement: S1 Table — (DOCX) [file pone.0134997.s003.docx]

**S1 Table. Confirmatory factor analysis**

| **Factor** | **Test** | **Time-point*** | **Factor loading†** |
| --- | --- | --- | --- |
| **Non-Verbal IQ** | | | |
| ***Pre-school*** | WPPSI Block Design | T1 | 0.720 |
|  |  | T2 | 0.760 |
|  | WPPSI Object Assembly | T1 | 0.709 |
| ***School*** | WISC Block Design | T4 | 0.861 |
|  |  | T5 | 0.807 |
|  | WISC Matrix Reasoning | T5 | 0.694 |
| **Speech** | | | |
| ***Pre-school*** | DEAP | T1 | 0.705 |
|  |  | T2 | 0.698 |
|  | PSrep | T1 | 0.753 |
|  |  | T2 | 0.851 |
| ***School*** | PSrep | T3 | 0.798 |
|  | NWrep | T3 | 0.851 |
|  |  | T4 | 0.896 |
|  |  | T5 | 0.839 |
|  | CNrep | T5 | 0.873 |
| **Language Grammar** | | | |
| ***Pre-school*** | CELF Sentence Structure | T1 | 0.659 |
|  |  | T2 | 0.747 |
| ***School*** | CELF Sentence Structure | T3 | 0.718 |
|  | CELF Word Structure | T4 | 0.725 |
|  | TROG T4 | T4 | 0.854 |
|  | CELF Recalling Sentences | T5 | 0.793 |
|  | CELF Formulated Sentences | T5 | 0.771 |
|  | TROG | T5 | 0.840 |
| **Language Vocabulary** | | | |
| ***Pre-school*** | CELF Expressive Vocabulary | T1 | 0.789 |
|  | ROWPVT | T2 | 0.753 |
| ***School*** | CELF Expressive Vocabulary | T3 | 0.835 |
|  |  | T4 | 0.871 |
|  | ROWPVT | T4 | 0.78 |
|  |  | T5 | 0.759 |
|  | CELF Expressive Vocabulary | T5 | 0.858 |
| **Literacy** | | | |
| ***T3*** | EWR | T3 | 0.957 |
|  | SWR | T3 | 0.965 |
|  | SPELLING | T3 | 0.834 |
| ***T4*** | EWR | T4 | 0.871 |
|  | SWR | T4 | 0.968 |
|  | SPELLING | T4 | 0.852 |
| **Phonology** | | | |
| ***Pre-school*** | PD | T2 | 0.795 |
|  | WR | T2 | 0.638 |
| ***School*** | PD | T3 | 0.775 |
|  |  | T4 | 0.904 |
|  |  | T5 | 0.786 |
| **Rapid Automatised Naming** | | | |
| ***Pre-school*** | RAN Objects | T2 | 0.867 |
|  | RAN Colours | T2 | 0.800 |
| ***School*** | RAN Objects | T3 | 0.814 |
|  |  | T4 | 0.842 |
|  |  | T5 | 0.779 |

**†**degree of correlation between the individual test score (initial raw score) and the final factor score *average ages at each time-point (years) are T1 = 3.8, T2 = 4.7, T3 = 5.7, T4 = 6.6, T5 = 8.1

WPPSI, Wechsler Preschool and Primary Scale of Intelligence; WISC, Wechsler Preschool and Primary Scale of Intelligence; DEAP, Diagnostic Evaluation of Articulation and Phonology; PSrep, Preschool Repetition Test; NWrep, Non Word Repetition Test; CNrep, Children's Test of Nonword Repetition; CELF, Clinical Evaluation of Language Fundamentals; TROG, Test for Reception of Grammar; ROWPVT, Receptive and Expressive One-Word Picture Vocabulary Tests; EWR, Early Word Recognition; SWR, Single Word Reading; PD, Phoneme Deletion; WR, Word Recall; RAN, Rapid Automatised Naming
