# Supplementary material for: Copy Number Variation Screen Identifies a Rare De Novo Deletion at Chromosome 15q13.1-13.3 in a Child with Language Impairment
Source: PLoS One. 2015 Aug 11;10(8):e0134997. doi: 10.1371/journal.pone.0134997 (PMC4532445; doi:10.1371/journal.pone.0134997)
Supplement: S2 Table — (DOCX) [file pone.0134997.s004.docx]

**S2 Table. Quantitative PCR primers**

| **CNV** | **Amplicon** | **Forward primer sequence** | **Reverse primer sequence** | **Size (bp)** |
| --- | --- | --- | --- | --- |
| **15q13.1-13.3 deletion** | 1 | 5'-CCG GTG AAC AGA GAG AGG AG | 5'-GCT TGG TGT GAG CAG CAT AA | 155 |
|  | 2 | 5'-TGG GGG TGC TGC TTC GTT CT | 5'-AGG CTG CCT GTG GGC ATC AA | 194 |
|  | 3 | 5’-CCC GCT CTT TGA ACA AAC CA | 5’-CAT TTC CGC TGG TCC TGA AG | 121 |
|  | 4 | 5’-TGG ATG GGT GGT AGG AAT GG | 5’-AGA GCT AAT GTC TCC GCC TC | 94 |
|  | 5 | 5’- TGG GCT CCT TCC ACT TGT AG | 5’- ATT GCA GAC TTC AGG CTC CT | 119 |
|  | 6 | 5'-TGG GAG GGC GTC CTG CTA AA | 5'-TCA TCC CAG CAA ACG CCG CT | 155 |
| **16q13.11**  **duplication** | 1 | 5’-TCC AGG CAC TTG ATG CTC TC | 5’-AAG CAA AGC TGC AAT CTG GC | 140 |
|  | 2 | 5'-TGT TGG GTC AGA GGG TCA TA | 5'-TGC TCA TAC CTA TTT GCA AAA CCC | 142 |
|  | 3 | 5'-GAT ATG TCA ACC TTA GGG CTG TTT | 5'-AAC AAC AAA GGG ATA ATA CTT CGG | 70 |
|  | 4 | 5'-ACC TTC TGG TGG ATC ACA GG | 5'-GCA CAG GCA GAT TCA AGA CA | 161 |
|  | 5 | 5'-GCA TGA CCC AAC AGA ATG AGC | 5'-AAA TGA AGG CAG CCA TGC AG | 94 |
|  | 6 | 5'-TGG TGG TGT TTG TTA GTG CCT | 5'-GCC CTC GTA TGG CAG C | 116 |
| ***SEMA3A* duplication** | 1 | 5'-GCT GGC CAC AAC ATT CAT T | 5'-GGT TGC TCA CAT TGA ACT GC | 153 |
|  | 2 | 5'-AGC ATA TCC AGG ACC ACA TAA AAT | 5'-GGA AGC GGT GGA CTA GTA AAG TAT AG | 164 |
|  | 3 | 5'-TTT ATA TAG GCC ACG CAG ACC | 5'-AGC TGT CCA TGT TCT GTT CTC A | 154 |
|  | 4 | 5'-CCT GCC GTG GAG TTA CAT TT | 5'-TCA AGC ACA CCA TCC AGA AA | 161 |
|  | 5 | 5'-GCT CAC ATG CCA GAG ACA AA | 5'-TCC CAG GTT GGT TGT TTC AT | 152 |
|  | 6 | 5'-ACA GCT CCA CAT CAC CAA CA | 5'-CCT TTG ACA GGC TGC ACA TA | 190 |
| **Normal-copy**  **reference** | | 5’- aaa tgc tgc aca gaa tcc ttg | 5’-gaa aag cat ggt cgc ctg tt | 44 |
