# Supplementary material for: Copy Number Variation Screen Identifies a Rare De Novo Deletion at Chromosome 15q13.1-13.3 in a Child with Language Impairment
Source: PLoS One. 2015 Aug 11;10(8):e0134997. doi: 10.1371/journal.pone.0134997 (PMC4532445; doi:10.1371/journal.pone.0134997)
Supplement: S3 Table — (DOCX) [file pone.0134997.s005.docx]

**S3 Table. Predicted CNV sizes**

|  | **Size (bp)** | | | **Confidence** | |
| --- | --- | --- | --- | --- | --- |
|  | **PennCNV** | **QuantiSNP** | **Merged** | **PennCNV*** | **QuantiSNP†** |
| **Mean** | 116,246 | 77,983 | 106,682 | 26 | 33 |
| **Max** | 1,856,390 | 828,101 | 1,856,389 | 1,144 | 682 |
| **Min** | 306 | 306 | 305 | 10 | 10 |

* Confidence value

† Maximum Log Bayes Factor
