# Supplementary material for: Copy Number Variation Screen Identifies a Rare De Novo Deletion at Chromosome 15q13.1-13.3 in a Child with Language Impairment
Source: PLoS One. 2015 Aug 11;10(8):e0134997. doi: 10.1371/journal.pone.0134997 (PMC4532445; doi:10.1371/journal.pone.0134997)
Supplement: S4 Table — (DOCX) [file pone.0134997.s006.docx]

**S4 Table. Size distribution of predicted CNVs**

| Mb | PennCNV | | QuantiSNP | | Merged* | |
| --- | --- | --- | --- | --- | --- | --- |
|  | n | % | n | % | n | % |
| <0.1 | 1879 | 59.3% | 2345 | 74.0% | 1968 | 62.1% |
| 0.1-0.2 | 922 | 29.1% | 709 | 22.4% | 873 | 27.5% |
| 0.2-0.3 | 210 | 6.6% | 77 | 2.4% | 191 | 6.0% |
| 0.3-0.4 | 58 | 1.8% | 20 | 0.63% | 60 | 1.9% |
| 0.4-0.5 | 24 | 0.76% | 7 | 0.22% | 28 | 0.88% |
| 0.5-0.6 | 32 | 1.01% | 7 | 0.22% | 20 | 0.63% |
| 0.6-0.7 | 5 | 0.16% | 1 | 0.03% | 5 | 0.16% |
| 0.7-0.8 | 3 | 0.09% | 1 | 0.03% | 5 | 0.16% |
| 0.8-0.9 | 7 | 0.22% | 2 | 0.06% | 5 | 0.16% |
| 0.9-1.0 | 10 | 0.32% | 0 |  | 2 | 0.06% |
| 1.0-1.1 | 0 |  | 0 |  | 0 |  |
| 1.1-1.2 | 12 | 0.38% | 0 |  | 7 | 0.22% |
| 1.2-1.3 | 5 | 0.16% | 0 |  | 3 | 0.09% |
| 1.3-1.4 | 0 |  | 0 |  | 0 |  |
| 1.4-1.5 | 0 |  | 0 |  | 0 |  |
| 1.5-1.6 | 1 | 0.03% | 0 |  | 1 | 0.03% |
| 1.6-1.7 | 0 |  | 0 |  | 0 |  |
| 1.7-1.8 | 0 |  | 0 |  | 0 |  |
| 1.8-1.9 | 1 | 0.03% | 0 |  | 1 | 0.03% |
| 1.9-2.0 | 0 |  | 0 |  | 0 |  |
| Total | 3169 | 100% | 3169 | 100% | 3169 | 100% |

*merged predictions are a consensus of PennCNV and QuantiSNP predictions
