# Supplementary material for: Copy Number Variation Screen Identifies a Rare De Novo Deletion at Chromosome 15q13.1-13.3 in a Child with Language Impairment
Source: PLoS One. 2015 Aug 11;10(8):e0134997. doi: 10.1371/journal.pone.0134997 (PMC4532445; doi:10.1371/journal.pone.0134997)
Supplement: S5 Table — (DOCX) [file pone.0134997.s007.docx]

**S5 Table. CNVs frequency**

|  | **Copy number** | **Common**  **n (%)** | **Rare**  **n (%)** | **Novel with low overlap**  **n (%)** | **Novel**  **n (%)** | **Total**  **n (%)** |
| --- | --- | --- | --- | --- | --- | --- |
| **Deletion** | 0 | 68  (2.1) | 3  (0.01) | 0 | 0 | 71  (2.2) |
|  | 1 | 202  (6.4) | 37  (1.2) | 34  (1.1) | 3  (0.1) | 276  (8.7) |
| **Duplication** | 3 or 4 | 1726  (54.5) | 700  (22.1) | 390  (12.3) | 6  (0.2) | 2822  (89.1) |
|  | Total | 1996  (63.0) | 740  (23.4) | 424  (13.4) | 9  (0.3) | 3169  (100) |
