# Supplementary material for: Copy Number Variation Screen Identifies a Rare De Novo Deletion at Chromosome 15q13.1-13.3 in a Child with Language Impairment
Source: PLoS One. 2015 Aug 11;10(8):e0134997. doi: 10.1371/journal.pone.0134997 (PMC4532445; doi:10.1371/journal.pone.0134997)
Supplement: S6 Table — (DOCX) [file pone.0134997.s008.docx]

**S6 Table. Raw scores for performance of Proband 62**

| **Construct** | **Test** | **Age at test in months** | | | | |
| --- | --- | --- | --- | --- | --- | --- |
|  |  | **43** | **51** | **61** | **73** | **92** |
| **Non-Verbal Ability[1]** | Block Design | 76 | 100 | - | 88 | 70 |
|  | Object Assembly | 100 | - | - | - | - |
|  | Matrix Reasoning | - | 76 | - | - | 76 |
| **Speech** | Consonants[2] | 40 | - | 68 | - | - |
|  | Vowels[2] | 97 | - | 97 | - | - |
|  | Phonemes[2] | - | - | 79 | - | - |
|  | Preschool non-word repetition[3] | 39 | 50 | 67 | - | - |
|  | Non-word repetition[4] | - | - | 20 | 40 | - |
| **Language[5]** | Expressive Vocabulary | 70 | 88 | 88 | 76 | 88 |
|  | Basic concepts | 76 | - | - | - | - |
|  | Receptive Vocabulary | - | 92 | - | 90 | 95 |
|  | Receptive Grammar | 76 | 94 | 76 | 65 | 55 |
|  | Formulating Sentences | 82 | - | - | - | 82 |
|  | Word Structure | - | - | - | 82 | - |
| **Literacy[6]** | Phonological awareness | 82 | - | - | 86 | - |
|  | Letter Knowledge | - | 84 | 106 | 110 | - |
|  | Single Word Reading (early words) | - | 98 | 88 | - | - |
|  | Single Word Reading | - | - | - | 104 | 104 |
|  | Passage Reading Accuracy | - | - | - | - | 106 |
|  | Reading Rate | - | - | - | - | 105 |
|  | Reading Comprehension | - | - | - | - | 105 |
|  | Spelling^4^ | - | - | - | - | 97 |
| **Number skills[7]** | Numerical Operations | - | - | - | - | 78 |

**References**

1. Wechsler D (2004) The Wechsler intelligence scale for children—fourth edition. London: Pearson Assessment.
2. Dodd B, Zhu H, Crosbie S, Holm A, Ozanne A (2002) Diagnostic evaluation of articulation and phonology (DEAP). London: Psychology Corporation.
3. Chiat S, Roy P (2007) The preschool repetition test: an evaluation of performance in typically developing and clinically referred children. J Speech Lang Hear Res 50: 429-443.
4. Gathercole SE, Willis CS, Baddeley AD, Emslie H (1994) The Children's Test of Nonword Repetition: a test of phonological working memory. Memory 2: 103-127.
5. Semel Em, Wiig EH, Secord W (1992) Clinical Evaluation of Language Fundamentals-Revised. San Antonio: Psychological Corporation.
6. Snowling MJ, Stothard S, Clarke P, Bowyer-Crane C, Harrington A, et al. (2009) YARC York Assessment of Reading for Comprehension Passage Reading.
7. Wechsler D (2005) Wechsler Individual Achievement Test Second UK Edition (WIAT-II UK). London: Psychological Corporation.
